# Ten year neurocognitive trajectories in first-episode psychosis

**DOI:** 10.3389/fnhum.2013.00643

**Published:** 2013-10-07

**Authors:** Helene E. Barder, Kjetil Sundet, Bjørn R. Rund, Julie Evensen, Ulrik Haahr, Wenche Ten Velden Hegelstad, Inge Joa, Jan O. Johannessen, Johannes Langeveld, Tor K. Larsen, Ingrid Melle, Stein Opjordsmoen, Jan I. Røssberg, Erik Simonsen, Per Vaglum, Thomas McGlashan, Svein Friis

**Affiliations:** ^1^Psychosis Research Unit/TOP, Division of Mental Health and Addiction, KG Jebsen Center for Psychosis Resarch, Oslo University HospitalOslo, Norway; ^2^Department of Psychology, University of OsloOslo, Norway; ^3^Vestre Viken Hospital TrustDrammen, Norway; ^4^Institute of Clinical Medicine, University of OsloOslo, Norway; ^5^Early Psychosis Intervention Centre, Psychiatry RoskildeRoskilde, Denmark; ^6^Regional Centre for Clinical Research in Psychosis, Psychiatric Division, Stavanger University HospitalStavanger, Norway; ^7^Department of Health Studies, University of StavangerStavanger, Norway; ^8^Institute of Psychiatry, University of BergenBergen, Norway; ^9^Psychiatric Research Unit, Psychiatry Roskilde, Department of Psychology and Educational Studies, Roskilde University and University of CopenhagenRoskilde, Denmark; ^10^Department of Behavioural Sciences in Medicine, University of OsloOslo, Norway; ^11^Department of Psychiatry, Yale University School of MedicineNew Haven, CT, USA

**Keywords:** neurocognition, longitudinal, first-episode psychosis, relapse, verbal memory

## Abstract

**Objective:** Neurocognitive impairment is commonly reported at onset of psychotic disorders. However, the long-term neurocognitive course remains largely uninvestigated in first episode psychosis (FEP) and the relationship to clinically significant subgroups even more so. We report 10 year longitudinal neurocognitive development in a sample of FEP patients, and explore whether the trajectories of cognitive course are related to presence of relapse to psychosis, especially within the first year, with a focus on the course of verbal memory.

**Method:** Forty-three FEP subjects (51% male, 28 ± 9 years) were followed-up neurocognitively over five assessments spanning 10 years. The test battery was divided into four neurocognitive indices; Executive Function, Verbal Learning, Motor Speed, and Verbal Fluency. The sample was grouped into those relapsing or not within the first, second and fifth year.

**Results:** The four neurocognitive indices showed overall stability over the 10 year period. Significant relapse by index interactions were found for all indices except Executive Function. Follow-up analyses identified a larger significant decrease over time for the encoding measure within Verbal Memory for patients with psychotic relapse in the first year [*F*_(4, 38)_ = 5.8, *p* = 0.001, η^2^ = 0.40].

**Conclusions:** Main findings are long-term stability in neurocognitive functioning in FEP patients, with the exception of verbal memory in patients with psychotic relapse or non-remission early in the course of illness. We conclude that worsening of specific parts of cognitive function may be expected for patients with on-going psychosis, but that the majority of patients do not show significant change in cognitive performance during the first 10 years after being diagnosed.

## Introduction

While cognitive deficits are frequently reported in first-episode psychosis (FEP) (Bilder et al., 2000; Addington et al., 2003; Kurtz, 2005), the longitudinal course remains an area of debate (Rund, 1998; Townsend and Norman, 2004). Most longitudinal studies of neurocognition in FEP refer to follow-up intervals of 2–5 years, describing stability or small improvements over time (Gold et al., 1999; Hoff et al., 1999; Hill et al., 2004; Rund et al., 2007). A few studies covering 10 or more years after first episode (Stirling et al., 2003; Hoff et al., 2005) or early onset schizophrenia (Oie et al., 2010) provide contrasting results ranging from overall stability to selective deterioration or developmental arrest.

Inconsistent findings are generally explained by methodological challenges, such as use of multiple test batteries assessing a variety of domains over different lengths of follow-up periods (Bozikas and Andreou, 2011). Further, treatment with antipsychotic medication may improve neurocognitive performance (Bilder et al., 2002) although the decrement from normal performance remains (Keefe et al., 2006). Thus, different types and effects of medication may confound interpretations of neurocognitive change.

Clinical subgroups with FEP most likely experience different neurocognitive trajectories that are concealed when the groups are merged and averaged over time. In an attempt to identify longitudinally emerging subgroups, cross-sectional studies have compared FEP- and multi-epsiode patients. Such studies tend to report lower performances in multi-episode samples (Pukrop et al., 2006; Braw et al., 2008; Sponheim et al., 2010).The cross-sectional method leaves open the possibility that group differences stem from incomplete sample matching (Rund, 1998; Moritz et al., 2002), in that well-functioning FEP patients drop out from mental health care follow-ups (Braw et al., 2008). Also, there is a risk of biased sample selection (Braw et al., 2008) in which poor outcome patients are selected to the multi-episode samples. To date, investigations of a relationship between neurocognitive course and recurrent psychotic episodes are not reported in studies of FEP patients with a longitudinal multi-assessment design. Consequently, it is still unclear whether cognitive dysfunctions remain stable, decrease, or fluctuate (Rodriguez-Sanchez et al., 2008), and if there are systematic differences between clinically defined subgroups (Knoll et al., 1998).

The early phase of psychosis has been referred to as a “critical period” in the course of illness, suggesting that when deterioration occurs, it proceeds aggressively in the first 2–3 years, with subsequent relative stability (Birchwood et al., 1998; Crumlish et al., 2009). Whether the hypothesis of a critical period also applies to the course of neurocognitive functions remains unknown.

Identifying and describing subgroups is a commonly used method in the effort to reduce heterogeneity and increase understanding of psychotic disorders and their progression. In terms of neurocognitive heterogeneity, there is an on-going debate on specific vs. more generalized cognitive impairments, especially surrounding illness onset (Lencz et al., 2006).

Verbal memory dysfunction is one of the most consistently reported cognitive deficits and among the best predictors of functional outcome in schizophrenia (Toulopoulou and Murray, 2004; Koutsouleris et al., 2012). Also executive function is reported to contribute in predicting transition to psychosis in at-risk patients (Chan et al., 2006).

However, it has been argued that specific effects are small compared to a generalized effect in schizophrenia (Dickinson et al., 2008).

Controversies on patterns and size of deficits may be interpreted in light of the aforementioned potential critical period; in which certain domains may develop into relatively more distinct deficit profiles, causing the degree of impairment to be highly influenced by the timing of assessment (Gonzalez-Blanch et al., 2006).

A recent meta-analytic review found that cognitive impairments for verbal learning and memory or encoding were greatest in the early phase of the illness (Mesholam-Gately et al., 2009). This is consistent with the reports by Heinrichs and Zakzanis (1998) and others (Cirillo and Seidman, 2003) who argue that if a selective or disproportionate cognitive deficit does exist at the “domain level” in schizophrenia, it would be in the domain of verbal declarative memory (Saykin et al., 1991; Mesholam-Gately et al., 2009; Kern et al., 2010; Bozikas and Andreou, 2011). Therefore, narrowing focus from global to more specific areas of neurocognition seems justified, and is of particular importance to this patient group.

Verbal memory impairment is reported to be a potential genetic marker of vulnerability in non-affected relatives (Skelley et al., 2008), and in high-risk individuals that subsequently transit to psychosis (Fusar-Poli et al., 2012; Giuliano et al., 2012). Further, verbal memory deficits indicate a more rapid conversion to psychosis (Seidman et al., 2010; Koutsouleris et al., 2012) through the prodromal period and the onset of a first-episode (Pukrop et al., 2007; Valli et al., 2012). Additionally, over the long-term, there appears to be some evidence of a further deterioration in verbal memory, contrasting a pattern of general neurocognitive stability over time (Bozikas and Andreou, 2011). Verbal learning is also related to insight (Buchy et al., 2010; Engh et al., 2011; Wiffen et al., 2012), and social functioning in psychotic illnesses (Stain et al., 2012), making it an area of high importance to clinical therapy and rehabilitation.

Thus, verbal memory and disease progression appear to be closely associated through the early phases of illness. This relationship may also be evident in a long-term perspective, possibly mediated through an early critical period.

To explore this hypothesis requires longitudinal studies of FEP patients, applying multiple assessments and detailed clinical and neurocognitive data.

In previous reports from the TIPS study (Rund et al., 2007; Barder et al., 2013) we concluded that the overall neurocognitive course remained stable during the first 2–5 years after illness onset. However, mild cognitive deterioration was observed in verbal learning and motor speed, and applied to patients having experienced more than one psychotic episode during the 5 year span compared to those with a stable remission of their index psychosis, i.e., a single episode and no re-occurring episodes Barder et al. (2013). In this study we ask if the same holds true over a 10 year period, based on recurrent episodes (relapses) of psychosis within the first year after treatment.

## Research questions

1. Does neurocognitive functioning change over the 10 year period from start of treatment in FEP patients?
2. Does illness severity (“Early relapse or no early relapse”) differentiate the longitudinal neurocognitive trajectory in FEP patients?
3. Does evidence support global or specific neurocognitive change related to illness severity over a 10 year follow-up period, and is verbal memory especially sensitive?

## Materials and methods

### The TIPS project

The present report originates from the Early Treatment and Intervention in Psychosis Study (TIPS), a prospective longitudinal study of the relationship between duration of untreated psychosis (DUP) and outcome in FEP. The study was carried out in four Scandinavian health care sectors; three in Norway (Oslo, Stavanger and Haugesund) and one in Denmark (Roskilde). The project has been approved by the Regional Committee for Medical Research Ethics Health Region II and Health Region East in Norway, and The regional committee for science ethics region Sjælland, Denmark. Informed consent was obtained from all participants.

A total of 301 patients between 15–65 years of age were included in the TIPS study. All patients met the DSM-IV criteria for non-organic psychosis, were actively psychotic without previously receiving adequate antipsychotic treatment at time of inclusion, and were included in a defined treatment program (Melle et al., 2004). Of the total group, 213 patients were older than17 years and available for neuropsychological testing after remission of the psychotic symptoms within the first 3 months (defined as a score lower than 4 on the relevant PANSS positive symptoms (Kay et al., 1987), or after 3 months when the patient gave consent and could cooperate, irrespective of remission. All patients were invited for reassessment at 1, 2, 5, and 10 year follow-ups (see Subjects for actual number of patients who met for testing) and comprise the 10 year follow-up study group.

### Measures

#### Clinical instruments

The structured clinical interview for the DSM-IV; SCID (First et al., 1995) was used for diagnostic purposes. Trained clinical research personnel carried out diagnostic evaluations. Symptom levels were assessed with the Positive and Negative Syndrome Scale; PANSS (Kay et al., 1987) and global functioning with the Global Assessment of Functioning Scale—split version (GAF).

DUP was measured as the time from the first onset of psychotic symptoms (defined as the first week with a PANSS score of 4 or more on one of the Positive scale items 1, 3, 5, 6, or General scale item 9) to the start of first adequate treatment of psychosis (defined as start of adequate antipsychotic medication or admission to hospital for treatment of acute psychosis).

Relapse was defined as the reappearance of positive psychotic symptoms (as defined above) for at least 7 days.

Premorbid functioning was measured using the Premorbid Adjustment Scale (PAS) (Cannon-Spoor et al., 1982). A previous analysis identified two premorbid dimensions: *social* consisting of PAS items social isolation and peer relationships and *academic* which comprise school performance and school adaptation (Larsen et al., 2004).

Drug and alcohol abuse for the period of 6 months prior to the start of treatment was assessed by the Alcohol and Drug Use Scale (Mueser et al., 2003).

Satisfactory inter-rater reliability was found with overall agreement for DSM-IV diagnostic categories at baseline, Kappa: 0.76. PANSS: ICC (1, 1): 0.88 for positive symptoms, 0.76 for negative symptoms, and 0.53 for general symptoms (Friis et al., 2003).

#### Neurocognitive measures

The subtests Similarities, Block Design and Digit Span from WAIS-R (Wechsler, 1981) were used to calculate an IQ-estimate at baseline.

The neurocognitive test battery was found to assess five separate domains validly, as identified in a factor analytic study of baseline performance (Friis et al., 2002). Between the 5- and 10 year assessments the test battery was slightly revised, and the present paper follows four of the five original indices over the 10-year follow-up interval. Two of these four indices; the Executive Functioning (EF) and Motor Speed (MS), are identical to the indices identified in the baseline factor analysis (Friis et al., 2002). The original Working Memory index (WM) is in the present study replaced by the Verbal Fluency index (VF), since the Controlled Oral Word Association Test (COWAT) (Spreen and Strauss, 1998) is the only subtest from the WM-index that is also represented at 10 year follow-up. Hence, the WM- index was re-defined as the Verbal Fluency index.

The EF-, MS-, and VF-indices consist of the same test versions at all follow-up assessments.

For the Verbal Learning index (VL-index), the revised version of the California Verbal Learning Test (CVLT) was used at 10 year follow-up; CVLT-II (Delis et al., 2004). The number of words and trials were identical to the original version used at the previous assessments, CVLT (Delis et al., 1987). Fusing raw scores obtained from these two test versions in the same analyses was justified since equivalency in total learning and long-delay free recall raw scores is reported in healthy individuals (Delis et al., 2000). The psychometric characteristics of the Norwegian translation of the original English CVLT–II have been retained (Bosnes, 2007), providing support for good equivalency in the two Norwegian CVLT versions as well. Since the Danish language is very close to Norwegian, this is assumed to hold true also for the Danish versions.

Thus, the only change in the test battery in the present study was the replacement of the CVLT-revised version between 5-and 10 year assessment.

The domain scores were calculated as the mean z-score of the tests included based on means and standard deviations of the total sample at baseline (*N* = 213) (Barder et al., 2013). (See Table 1).

**Table 1:** **The four neurocognitive indices with the corresponding subtests and raw scores at each time point for the follow-up sample**.

	**Baseline**		**One year**		**Two year**		**Five year**		**Ten year**		**ANOVA**	
	***M***	***SD***	***M***	***SD***	***M***	***SD***	***M***	***SD***	***M***	***SD***	***F*_(4, 39)_**	***P***	**η^2^**
Verbal learning—index (VL)	0.25	(0.7)	0.28	(0.78)	0.31	(0.8)	0.20	(0.7)	−0.13	(0.9)	2.2	0.089	0.2
CVLT total immediate recall (learning)	56.2	(10.5)	56.4	(13.6)	57.5	(13.5)	56.6	(10.8)	49.9	(12.7)			
CVLT delayed free recall	13.0	(2.4)	13.0	(2.6)	12.7	(3.1)	12.7	(2.6)	11.7	(3.4)			
CVLT mean errors at recall	0.35	(0.6)	0.29	(0.5)	0.26	(0.4)	0.42	(0.6)	0.47	(0.8)			
Motor speed—index (MS)	0.03	(0.8)	0.10	(0.8)	0.19	(0.7)	−0.11	(0.6)	−0.01	(0.6)	1.8	0.145	0.2
FTT (mean score for the two hands)	48.3	(6.7)	49.0	(7.0)	50.0	(6.0)	47.1	(5.5)	48.0	(5.4)			
Executive functioning—index (EF)	0.13	(0.8)	0.22	(0.8)	0.19	(0.9)	0.31	(0.6)	0.21	(0.9)	1.1	0.372	0.1
WCST categories completed	5.3	(1.3)	5.5	(1.4)	5.5	(1.4)	5.6	(0.9)	5.6	(1.5)			
WCST perseverative responses	13.6	(13.0)	11.3	(10.0)	12.0	(12.7)	8.9	(6.5)	9.7	(6.4)			
WCST attempts to first category	19.0	(14.8)	19.4	(19.4)	19.4	(20.6)	20.4	(20.7)	23.2	(26.5)			
Verbal fluency—index (VF)	0.22	(0.7)	0.18	(0.9)	0.31	(1.1)	0.55	(0.86)	0.41	(1.3)	3.0	0.028	0.2
COWAT (sum of F-, A-, and F-words)	34.2	(8.1)	33.8	(10.3)	35.2	(12.0)	37.8	(9.3)	36.3	(14.6)			

CVLT, California Verbal Learning Test (Delis et al., 1987, 2004); FTT, Finger tapping test (Lezak, 1995); WCST, Wisconsin Card Sorting Test, (128 cards computer version) (Heaton et al., 1993); COWAT, Controlled Oral Word Association task (Spreen and Strauss, 1998).

### Subjects

Two hundred and thirteen patients between 18 and 65 years of age were assessed at baseline. Of these, 135 volunteered for re-testing at 1 year follow-up, and of these, 105 at 2 year follow-up. From this sample, 62 were tested for the fourth time at the 5 year follow-up, and are described in Barder et al. (2013). Of the 62 patients at 5 year follow-up, 43 patients were re-tested at 10 year follow-up. Thus, the group of 43 patients with valid data from all five assessments spanning the 10 year follow-up period were included in the present study. This sample is referred to as the follow-up sample (*n* = 43). The group of patients who missed at least one of the five assessments will be referred to as the remaining sample (*n* = 170). The present sample includes all those taking part in the 5 year follow-up study (Barder et al., 2013), except for 19 subjects lost between the 5 and 10 years follow-up.

Baseline demographic and clinical characteristics for the two samples are presented in Table 2.

**Table 2:** **Demographic variables and symptom scores at baseline**.

**Variable**	**Follow-up sample (*n* = 43)**	**Remaining sample (*n* = 170)**	**χ^2^***/t/z*	***p***
Age (M, SD)	27.8 (8.8)	28.3 (10.1)	*t*_(211)_ = 0.3	0.787
Gender (Male)	22 (51%)	101 (59%)	χ^2^_(1, 213)_ = 0.4	0.421
Education (M, SD)	13.1 (2.9)	11.7 (2.2)	*t*_(205)_ = 3.1	0.003
IQ estimate (M, SD)	102.9 (9.0)	97.9 (10.6)	*t*_(208)_ = 2.9	0.005
DUP^1^ (weeks) (Median, Range)	7 (0–174)	11 (0–966)	*z* = 1.0	0.338
PAS social, childhood (M, SD)	0.8 (0.9)	1.0 (1.1)	*t*_(211)_ = 1.2	0.241
PAS social, change (M, SD)	1.0 (1.4)	0.8 (1.5)	*t*_(211)_ = 1.0	0.365
PAS academic, childhood (M, SD)	1.5 (1.1)	1.8 (1.3)	*t*_(211)_ = 1.1	0.278
PAS academic, change (M, SD)	0.7 (1.4)	0.7 (1.2)	*t*_(211)_ = 0.0	0.970
PANSS Positive score	20.4 (5.1)	20.2 (5.8)	*t*_(210)_ = 0.2	0.860
PANSS Negative score	13.8 (7.4)	15.6 (6.7)	*t*_(210)_ = 1.5	0.122
PANSS General score	34.1 (9.3)	35.6 (10.1)	*t*_(209)_ = 0.9	0.375
GAF-Function score	30.8 (9.4)	32.4 (10.7)	*t*_(209)_ = 0.9	0.374
GAF-Symptom score	28.7 (7.0)	29.7 (7.1)	*t*_(209)_ = 0.8	0.403
Alcohol abuse (N, %)	2 (4.6%)	24 (14.3%)	χ^2^_(1, 212)_ = 2.9	0.088
Drug abuse (N, %)	8 (18.6%)	39 (23.1%)	χ^2^_(1, 212)_ = 0.4	0.528
**DIAGNOSE, (DSM-IV)**				
Schizophrenia	10 (23.3%)	47 (27.6%)		
Affective psychosis w/ mood incongruent symptoms	5 (11.6%)	24 (14.1%)		
Schizoaffective	5 (11.6%)	21 (12.4%)		
Schizophreniform	13 (30.2%)	38 (22.4%)	χ^2^_(6, 213)_ = 4.3	0.637
Delusional disorder	2 (4.7%)	13 (7.6%)		
Brief psychotic episode	5 (11.6%)	9 (5.3%)		
Psychotic disorder NOS	3 (7.0%)	18 (10.6%)		

1 Duration of untreated psychosis.

The follow-up sample consisted of equal numbers men and women, they were in their late twenties, and had an estimated average IQ of around 100. Symptom ratings (PANSS) were severe at baseline but rated as mild from 3 months of treatment to follow-up (data not shown). Significant, differences between the two samples were found on two baseline measures: the follow-up sample had slightly longer education and higher IQ-estimate than the remaining sample. Hence, education and IQ were used as correcting covariates in follow-up analyses in order to increase the representativeness of our sample to the total group of TIPS patients. The diagnostic distribution did not differ significantly between groups at any of the follow-ups.

## Groups defined by presence of relapses

The follow-up sample was divided into two groups based on the number of relapses experienced by the individuals during their first year of treatment; no early relapses (*N* = 31), and one to three early relapses” (including 3 continuously psychotic patients) (*N* = 12), hereafter referred to as the “No early relapse-group” and the “Early Relapse-group,” respectively.

The groups did not differ significantly on demographic variables (age, gender, estimated IQ, years of education), nor in childhood academic or social function, or premorbid change in function (PAS), but the No early-relapse group was characterized by a significantly higher symptom load as measured by the PANSS positive [21.7 vs. 16.9, *t*_(41)_ = 3.0, *p* < 0.004] and PANSS negative symptom scores [15.4 vs. 9.5, *t*_(41)_ = 2.46, *p* = 0.018] at baseline.

The No early relapse-group also performed significantly better on the VF-index at baseline [0.37 vs. −0.16, *t*_(41)_ = 2.22, *p* = 0.032], but no group differences were found for the other neurocognitive indices.

The Early relapse- group had significantly more patients with a narrowly defined schizophrenia diagnosis (schizophrenia, schizophreniform, or schizoaffective) at baseline, compared to the No early-relapse group [χ^2^_(1, 43)_ = 5.2, *p* = 0.023].

In order to identify discrepancies in the distributions of scores between the total sample and the follow-up sample, a screening of the neurocognitive indices in the total sample (the remaining sample and the follow-up sample) divided by “relapse or not first year” was carried out. The screening illustrated a pattern of group differences between the two “relapse groups” consistent with the results from the follow-up sample over time. The differences between the “early relapse-group” and the “no-early relapse group” were slightly more pronounced in the total sample. Thus, the group differences found in the follow-up sample are assumed to be a conservative estimate of differences present in the larger sample.

The follow-up sample was also divided into “Relapse or no relapse” based on the first 2 years (17 vs. 26), and the first 5 years (26 vs. 17), in order to analyze if any of these groupings would be better in distinguishing between the two groups' 10 year neurocognitive trajectories.

## Attrition/missing data

The four index scores consisted of a total of 8 subtests. In cases of missing data, the group mean was inserted. This applied for less than 4% of the follow-up sample at the first four time points, and for 6% of the sample at the 10 year follow-up. The four index scores were calculated after missing scores were replaced by valid group mean score obtained at the given time point.

## Medication

At the 10 year follow-up assessment 31 patients were using antipsychotic medication, and 12 patients were not. There was no significant difference in continued medication between the early relapse—and the no early-relapse group at 10 year follow-up [83 vs. 68%, respectively, χ^2^_(1, 43)_ = 1.0, *p* = 0.307].

## Statistical analysis

Analyses were conducted using the statistical package SPSS (PAWS) for Windows (version 18). Group differences for continuous variables were evaluated with analyses of variance and *t*-tests. Chi-square tests were used for categorical variables.

A within-group repeated measure multiple analysis of variance, MANOVA, was performed to investigate the neurocognitive development over time (five assessments) with the four neurocognitive indices as dependent variables.

Four separate One-Way repeated measures ANOVAs were conducted, one for each of the neurocognitive indices, to analyse change over time.

Follow-up analyses of co-variance (MANCOVA, ANCOVAs), were performed in order to control for the effect of IQ and education over time (variables that differed between the follow-up and the remaining sample).

The hypothesis of an association between neurocognitive development and presence of relapse(s) was examined by a second set of repeated measure MANOVAs and ANOVAs with follow-up MANCOVAs and ANCOVAs, with “No relapse group”/“Relapse group” as the between-subject factor, and indices and time as the within-subject factors. Three sets of relapse or no relapse groups were defined based on relapse or not within the first year, the second year, or the fifth year.

Additional repeated measure ANOVAs were conducted on each of the three subtests that constituted the VL-index to investigate whether there was a differential relationship to first year relapse contained in the index score. Follow-up analyses were performed to control for covariates.

Bonferroni corrections were used to control for multiple comparisons.

## Results

Results for the eight tests and four indices over the five assessments are shown in Table 1. No statistically significant effect of assessment time was found on the set of the four neurocognitive indices [λ = 0.89, *F*_(4, 39)_ = 1.2, *p* = 0.329, η^2^ = 0.11], nor did the indices differ significantly from each other irrespective of time [λ = 0.93, *F*_(3, 40)_ = 1.0, *p* = 0.401, η ^2^ = 0.07]. However, a significant effect was found for the interaction between neurocognitive indices and time [λ = 0.47, *F*_(12, 31)_ = 2.9, *p* = 0.007, η^2^ = 0.53].

When analysing change in performance over time for each index, we found a significant effect of time for Verbal Fluency only; performance increased linearly from baseline to the 10 year follow-up. A near significant curvilinear (quadratic) development was found for Verbal Learning, where performance remained unchanged from baseline until the 2 year assessment and decreased progressively at the 5 and 10 year assessments.

**Figure 1:**
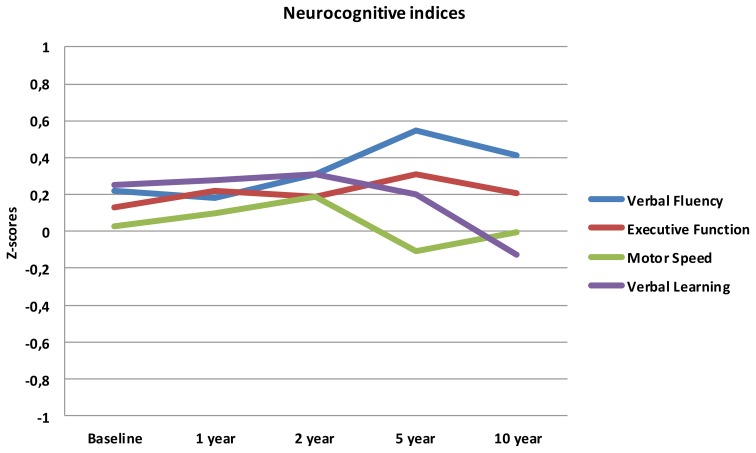
**Neurocognitive indices from baseline to 10 year follow-up**.

The mean scores over time for the four neurocognitive dimensions are displayed in Figure 1.

After controlling for IQ and education, the effect of assessment time and indices remained non-significant, but the interaction effect between time and indices lost its significance [λ = 0.58, *F*_(12, 28)_ = 1.7, *p* = 0.123, η^2^ = 0.42]. Since the effect size is still classified as large, the non-significant finding can be attributed to low statistical power due to small number of subjects within each group.

When analysing the effect of relapses during the first year on neurocognitive performance over the 10 year follow-up, a significant three-way interaction was found (Time × Indices × Relapse group), in addition to all two-way interactions and main effect of relapse-group (see Table 3). When controlling for IQ and education, the three-way interaction remained significant [*F*_(12, 27)_ = 2.2, *p* = 0.046, η ^2^ = 0.49].

**Table 3:** **Results from MANOVA; effects of early relapse on neurocognitive indices over time**.

**MANOVA**	***F***	***df***	***p***	**η^2^**
Time	0.2	4.38	0.925	0.02
Indices	0.5	3.39	0.686	0.04
Relapse(s) first year^1^	4.6	1.41	0.038^*^	0.10
Time **×** Indices	2.8	12.30	0.010^**^	0.53
Time **×** Relapse(s) first year	4.3	4.38	0.006^**^	0.31
Indices **×** Relapse(s) first year	5.7	3.39	0.002^**^	0.31
Time **×** Indices **×** Relapse(s) first year	2.8	12.30	0.010^**^	0.53

1 Total relapses first year: “No relapse first year” (N = 31), “Relapse(s) first year” (N = 12).

* p<0.05;

** p ≤ 0.01.

Analyses addressing the association between neurocognitive course and relapses within the first 2-, and 5-years, respectively, gave the following results; the 2-year grouping gave a significant interaction between indices and time [*F*_(12, 30)_ = 2.9, *p* = 0.009, η ^2^ = 0.50], and indices and relapse groups [*F*_(3, 39)_ = 4.1, *p* = 0.012, η ^2^ = 0.20]. The 5-year grouping gave a significant interaction between indices and time [*F*_(12, 30)_ = 2.8, *p* = 0.012, η ^2^ = 0.50], and a main effect of group [*F*_(1, 41)_ = 10.5, *p* = 0.002, η ^2^ = 0.20]. However, no significant interactions were found between time and relapse-groups, or any three-way interactions, as was the case for grouping with relapses first year as shown in Table 3.

**Figure 2:**
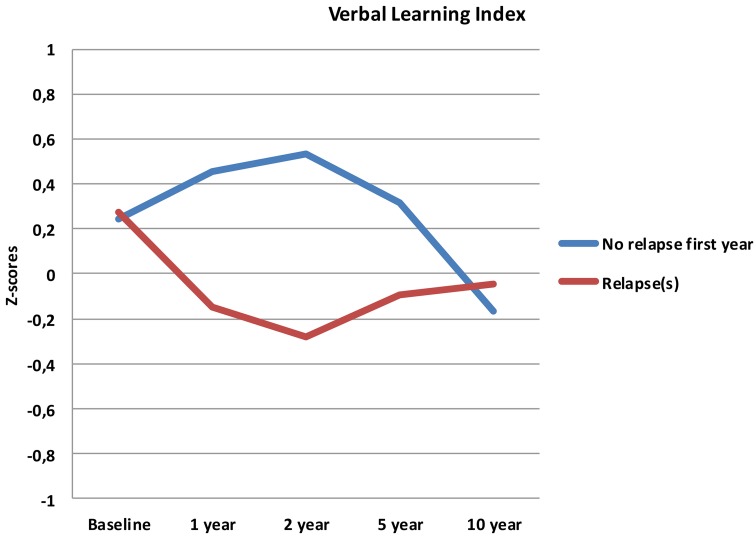
**Verbal Learning index from baseline to 10 year follow-up, split by early relapse**.

**Figure 3:**
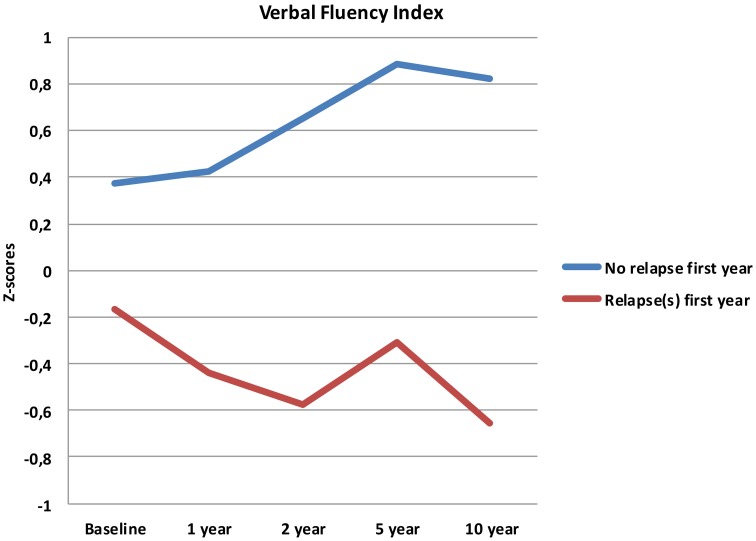
**Verbal Fluency index from baseline to 10 year follow-up, split by early relapse**.

**Figure 4:**
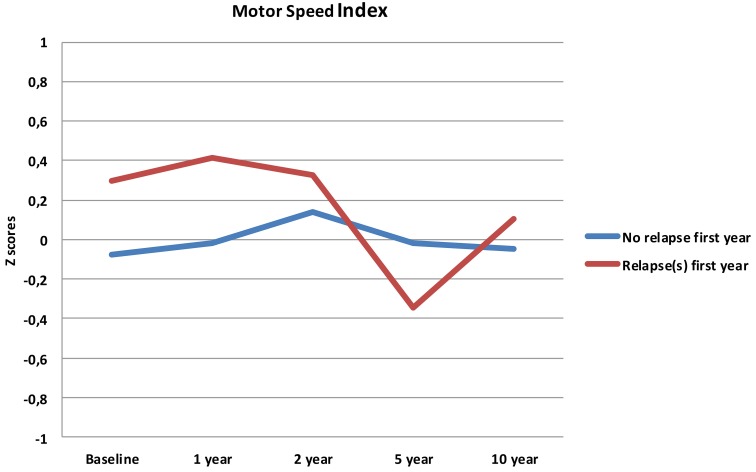
**Motor Speed index from baseline to 10 year follow-up, split by early relapse**.

Follow-up analyses with separate repeated measures ANOVA with “relapse(s) first year” as the between-subjects variable revealed significant interactions between relapse and all neurocognitive indices except Executive Function. For Verbal Fluency the no early-relapse group performed better than the early relapse-group at all time points, and for Verbal Learning significantly so at the 1 and 2 year follow-ups (see Figures 2–4).

After controlling for IQ and education the result remained significant for Verbal Learning and Verbal Fluency.

Because the VL-index consists of three subtests from the CVLT (see Table 1), further analyses were conducted to investigate if any of these were more strongly related to relapses first year. A significant change over time was identified for the total learning score over five trials [*F*_(4, 38)_ = 5.8, *p* = 0.001, η2 = 0.40]. The total learning score decreased from baseline to 10 years, with a significant main effect of relapse-group and a time by relapse-group interaction, indicating that the group with one or more relapses within the first year had a more prominent decline over the 10 year follow-up interval. A significant interaction between time and relapse-group was also found for the delayed free recall subtest [*F*_(4, 38)_ = 4.6, *p* = 0.002, η 2 = 0.35], but no main effect of group. No significant effects were found for mean recall errors. The results remained significant after controlling for IQ and education.

## Discussion

There are few studies investigating the relationship between neurocognitive and clinical variables over time, and studies addressing specific neurocognitive areas that may help differentiate this relationship are virtually absent. We aimed to investigate these issues in a well described sample of forty-three FEP patients followed over five assessments spanning 10 years.

Our first finding demonstrates overall neurocognitive stability over the 10 year follow-up period. The current study is one of the longest follow-up studies to date of FEP patients, and is supporting evidence of gradual cognitive stabilization in FEP over time (Bozikas and Andreou, 2011). However, as gradual stabilization is a group effect and may obscure substantial heterogeneity across individuals, we hypothesized that separating the sample based on presence of one or more relapses or non-remission of psychosis could differentiate the neurocognitive course. This hypothesis was supported for relapses within the first year, as the two groups showed different trajectories in three of the four neurocognitive indices over the 10 year follow-up period (see Figures 2–4). After controlling for IQ and education the group difference remained significant for Verbal Learning and Verbal Fluency, indicative of a specific neurocognitive change related to illness severity.

Analyses of relapse or not within the first 2- or 5-year follow-ups did not demonstrate the same differentiating effect for the neurocognitive trajectories. Psychotic relapse early on in the illness serves as the most potent prediction of neurocognitive deterioration over time.

The findings are in accordance with our previous report from the 5-year follow-up of the current patient sample (Barder et al., 2013) where numbers of relapses within the first 5 years after start of treatment were significantly related to a decrease in the VL-index over time. Conversely, subjects with no re-occurring episodes did not experience this decline. Based on the findings in the current study, a long term interaction between symptomatology and neurocognition appears to be present already 1 year after treatment initiation.

One of the few longitudinal studies of FEP reported a relationship between neurocognition and psychotic symptoms in the first 4–5 years (Hoff et al., 1999), but no associations using difference scores from baseline to 10 years follow-up (Hoff et al., 2005). The authors suggested that improvement in symptomatology may have a greater effect on cognitive abilities earlier in the illness but that its effects are diminished over time (Hoff et al., 2005).

Findings in the present study may be said to contradict such an hypothesis, as early remission of positive symptoms (no relapse) is associated with better neurocognitive performance at all follow-up assessments, and at 10 years follow-up for certain areas of neurocognitive functioning. On the other hand, if the concept of a critical period (Birchwood et al., 1998) is applied in this context, a long-term relationship between early relapse and neurocognitive course may be manifested through a sensitive first year period. Thus, early reduction in symptomatology (illness severity) may have a greater direct effect on neurocognition early in the illness, which is then mediating the subsequent long-term neurocognitive course.

Based on these premises, it is tempting to conclude that early re-occurrence of psychotic episodes may affect the neurocognitive course both earlier in the illness [as found in Barder et al. (2013)] and over as long as 10 years after start of treatment, at least for some neurocognitive areas. Moreover, neurocognitive dysfunction has recently been found to be more related to history of psychosis than to diagnostic category among bipolar and schizophrenia patients (Simonsen et al., 2011), also supporting a relationship between psychosis and neurocognitive functioning over time.

Some detailed explanation of the mechanisms behind this hypothetical process would presumably involve advanced measures of both brain structures and networks, which lies beyond the area of neurocognition and the scope of this study. However, one might speculate that recurring episodes early in the course of illness may serve as a vulnerability factor, leading toward a potentially vicious circle of truncation of education and employment, psychosocial challenges, more medication and medication non-adherence, which together may increase the risk of new psychotic episodes.

The method applied in this paper implies associations and does not allow a conclusion based on causality. Cognitive impairment could be the cause rather than the consequence of poorer clinical course. Nonetheless, detecting a relationship between relapse in the early phase and the long-term neurocognitive development may have significant clinical value for specific subgroups of patients. Hence, the findings may be taken as support for the relevance of early detection teams, as identifying subgroups with a possible vulnerability to neurocognitive impairments would be essential for rehabilitation and treatment programs.

In the present study, the relationship between early relapse and the continuing neurocognitive course was nuanced by the finding of a differentially stronger association for one subtest in the VL-index. Analysing the three subtests separately identified the encoding stage as the strongest factor related to early relapse. This indicates a differential relationship between psychosis and specific areas of neurocognition. Similar hypotheses have been proposed previously, through the concept of cognitive endophenotypes (Bilder et al., 2000; Barrett et al., 2009). In the present study, verbal learning is indicated as a relatively more vulnerable neurocognitive function in terms of associations with early recurrent psychotic episodes. These results are also in agreement with studies reporting that a lower performance on verbal memory was found to identify individuals with FEP with a poor outcome after 6 months of treatment (Bodnar et al., 2008).

There is, however, some controversy surrounding findings in this realm. For example, a meta-analysis assessing 70 studies of patients with schizophrenia found that clinical variables such as duration of illness, severity of psychopathology, and positive symptoms did not appear to influence the magnitude of memory impairment. Thus, the memory impairment was found to be of a considerable robustness and not readily moderated by variables that may seem relevant (Aleman et al., 1999).

Although these are compelling data, the robust nature of memory impairments in psychotic disorders is not fully identified until the long-term perspective is better understood. This is a very comprehensive task, and not likely to be answered on the basis of cross-sectional studies alone. In addition, the relationship between memory and clinical variables is inevitably complex due to the large heterogeneity in the FEP population e.g., (Lindsberg et al., 2009). Hence, it is of considerable interest to identify subgroups with weaker neurocognitive performance in order to adapt treatment and rehabilitation efforts accordingly. A possible sensitive period around first year after treatment initiation may give valuable information on this matter.

In the present paper we investigated if the presence of early relapses could differentiate longitudinal cognitive trajectories. In answering this question, one of the most relevant neurocognitive domains may be the verbal memory domain in general and the encoding stage in particular. This is consistent with a thorough review of verbal memory dysfunctions in schizophrenia, also reporting that the “memory” deficit appears primarily to be a learning impairment, and not merely the result of a problem with retrieval. Thus, it appears clear that the primary deficit is during the encoding stage of memory formation (Cirillo and Seidman, 2003).

Whether the decline in the present study has a clinical relevance is another question. The change in raw scores may not be clinically significant for the average follow-up sample, but the change between the relapse-groups may translate to a clinical level. Further, these findings are important in that they contrast a general notion of a clear improvement or a stable long-term course of neurocognition in FEP. A comparison between the relapse- and the no-relapse group at 2 year follow-up (the point of maximum discrepancy between groups), showed a difference of 16 words in favor of the no-relapse group. Although this discrepancy decreases again over time (Figure 2), we find it interesting for several reasons; the foundation for treatment adherence is often set in the first years after start of treatment (Masand et al., 2009). We would assume that for clinical therapy, and perhaps also for antipsychotic medication, the ability to adhere to, and benefit from treatment may be reduced in patients experiencing difficulties with encoding and memory of verbal auditory information. The issue of clinical significance is multifaceted, and thus, experienced clinical implications should not be ruled out.

Memory impairments are by no means exclusive to psychotic disorders, but are found in a range of other psychiatric illnesses, e.g., in major depressive disorder (Bora et al., 2009). Some studies have compared memory performance in clinical groups with major depression and schizophrenia, indicating impaired memory and especially impaired acquisition, as a particularly sensitive indication of schizophrenia, also after controlling for IQ and clinical symptom load (Egeland et al., 2003). Findings in the present study are in accordance with this, as the “Early-relapse”-group consisted of significantly more patients with a narrowly defined schizophrenia diagnosis at baseline. Such findings help emphasize, albeit indirectly, that verbal learning and memory may be key areas for further investigations of the long term neurocognitive course in psychotic illnesses.

In a longitudinal design several limitations need to be discussed, and possible re-test effects are a relevant concern in this regard. However, the relatively different trajectories found between the two groups reduce the likelihood of a strong re-test effect.

The degree of neurocognitive change is generally based upon comparison to patients' baseline performance. In the present study the patients were assessed after remission of the psychotic symptoms, or after 3 months, resulting in a relatively low level of symptoms at the time of the first test. This definition of baseline is likely to result in better neurocognitive performance compared to a baseline defined several weeks earlier. Thus, the broad definition of baseline applied in this study may have contributed to our findings of stability at group level, instead of a small increase as is reported in some studies (Gold et al., 1999).

A key aspect in the present study is to challenge the notion of a “group level” and explore subgroups that may show different neurocognitive trajectories. The question of whether there are subgroups with divergent paths embedded in a larger sample is not necessarily dependent upon a healthy control group for comparison; the patients' first assessment constitutes the reference point, which is followed-up along with assessments of the clinical development.

However, another point should be noted regarding the representativeness of the early relapse group. This group is small (*n* = 12), and had significantly lower PANSS positive and PANSS negative symptom scores at baseline (See Groups Defined by Presence of Relapse), indicating better functioning in terms of symptom load. Although this implies caution regarding direct generalization to a larger population, our results may in fact underestimate the magnitude of true differences between relapse and no-relapse samples over time.

The No early-relapse group had a significantly shorter DUP than the early relapse-group. This indicates a potentially large variation in the definition of “early phase” clinical characteristics used throughout the paper. However, a previous report from the TIPS-study found no significant association between DUP and neurocognitive functioning in the larger sample (Rund et al., 2004).

The follow-up sample in the present study had a significantly higher IQ and one more year of education than the remaining sample at baseline. Although results remained significant despite controlling for these variables in analyses, the follow-up sample is a relatively high-functioning sample in terms of cognition. Thus, conclusions regarding the larger population are not directly transferable, and must be drawn with caution. Yet, this implies that a more representative sample of early/no-early relapse patients may possibly demonstrate a more distinct neurocognitive discrepancy over time.

Due to lack of a control group we utilized comparisons to normative data to evaluate the (possible) effects of aging. For the VL-index (CVLT and CVLT-II tests), the expected change in standard norms is small or non-existent for both genders, suggesting that no (or very limited) age-related decline is expected.

## Conclusion

The results of our study have demonstrated overall 10 year neurocognitive stability after the start of treatment of FEP. Further, our findings identify possible neurocognitive subgroups based on early recurrent psychotic episodes, and support previous research identifying verbal memory as a neurocognitive function that is relatively more vulnerable to the effects of psychosis. Even if the design in this study does not allow drawing causal inferences, we have dissociated the encoding stage (acquisition of a list of words) as the only subtest from the VL-index showing a significant decrease over time. The relationship between verbal memory deficits and psychosis has been widely documented in the early stages of psychotic illness, but the longitudinal development of verbal memory in relation to clinical characteristics, is not yet clear. It is relevant to note that amongst papers examining FEP and neurocognition longitudinally, a large subset conclude on trends of stability, without investigating subgroups that may show significant change over time. Thus, both early illness severity, as measured by the presence of relapses, and the arena of verbal learning and memory, may be important factors in this regard.

### Conflict of interest statement

The authors declare that the research was conducted in the absence of any commercial or financial relationships that could be construed as a potential conflict of interest.
