# Corrigendum: “Ten year neurocognitive trajectories in first-episode psychosis”

**DOI:** 10.3389/fnhum.2014.00319

**Published:** 2014-05-19

**Authors:** Helene E. Barder, Kjetil Sundet, Bjørn Rund, Julie Evensen, Ulrik Haahr, Wenche Hegelstad, Inge Joa, Jan O. Johannessen, Johannes Langeveld, Tor K. Larsen, Ingrid Melle, Stein Opjordsmoen, Jan I. Røssberg, Erik Simonsen, Per Vaglum, Thomas McGlashan, Svein Friis

**Affiliations:** ^1^NORMENT, KG Jebsen Centre for Psychosis Research, Oslo University HospitalOslo, Norway; ^2^Department of Psychology, University of OsloOslo, Norway; ^3^Division of Mental Health and Addiciton, Institute of Clinical Medicine, Oslo University HospitalOslo, Norway; ^4^Psychiatry Roskilde, Early Psychosis Intervention CentreRoskilde, Denmark; ^5^Psychiatric Division, Regional Centre for Clinical Research in Psychosis, Stavanger University HospitalStavanger, Norway; ^6^Department of Health Studies, University of StavangerStavanger, Norway; ^7^Department of Clinical Medicine, Institute of Psychiatry, University of BergenBergen, Norway; ^8^Psychiatric Research Unit, Department of Psychology and Educational Studies, Roskilde University and University of ConpenhagenRoskilde, Denmark; ^9^Department of Behavioural Sciences in Medicine, University of OsloOslo, Norway; ^10^Department of Psychiatry, Yale University School of MedicineNew Haven, CT, USA

**Keywords:** neurocognition, psychosis spectrum disorders, first-episode, longitudinal studies, neuropsychiatry

A small part of the data on five of the 43 patients was accidentally displaced in the data file, which affected two of the results presented in the original manuscript.

The statistical significance of the MANCOVA (controlling for IQ and education) is not statistically significant. However, the effect size is still substantial (η^2^ = 0.444). The Motor Speed index does not show a statistically significant interaction with relapse-group, leaving Figure 4 redundant. A few minor changes in the decimals are added to Tables 1, 3.

**Table 1:**
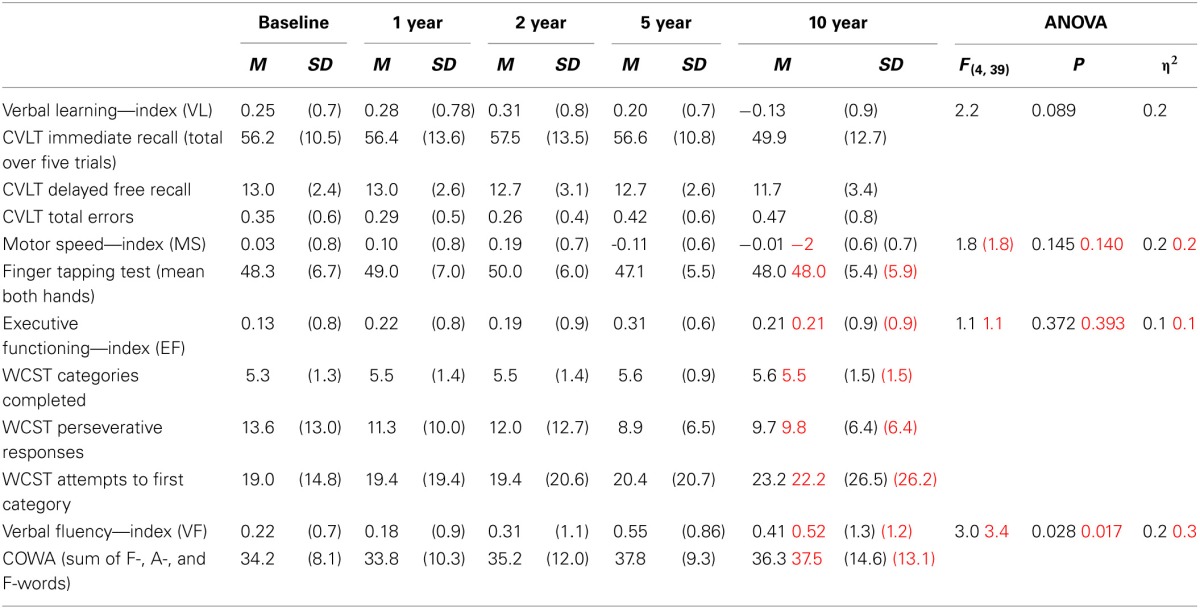
**The four neurocognitive indices with the corresponding subtests and raw scores at each time point for the follow-up sample (Corrected numbers in red)**.

CVLT, California Verbal Learning Test (Delis et al., 1987, 2004); FTT, Finger tapping test (Lezak, 1995); WCST, Wisconsin Card Sorting Test (Heaton et al., 1993); COWAT, Controlled Oral Word Association task (Spreen and Strauss, 1998).

**Table 3:**
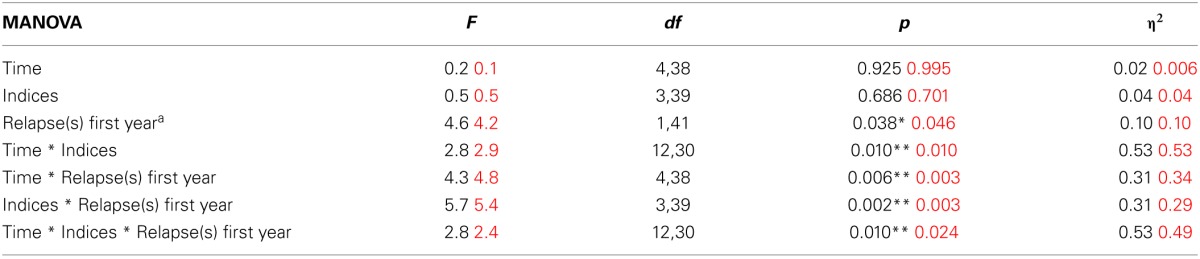
**Results from MANOVA; effects of early relapse on neurocognitive indices over time (Corrected numbers in red)**.

^a^Total relapses first year: “No relapse first year” (N = 31), “Relapse(s) first year” (N = 12).

## Conflict of interest statement

The authors declare that the research was conducted in the absence of any commercial or financial relationships that could be construed as a potential conflict of interest.

